# Better Lucky Than Clever

**DOI:** 10.1016/j.jaccas.2023.101975

**Published:** 2023-08-10

**Authors:** Pedro Custódio, Mark Westwood

**Affiliations:** a St Bartholomew’s Hospital, London, United Kingdom; b Hospital Vila Franca de Xira, Vila Franca de Xira, Portugual

**Keywords:** advanced cardiac imaging, cardiac magnetic resonance, clinical history

## Abstract

With an ever-expanding field of advanced cardiac imaging, clinicians tend to underestimate the importance of a detailed clinical history in reaching the correct diagnosis. This case illustrates 1 such example. (**Level of Difficulty: Intermediate.**)

## Central Illustration

**Figure:**
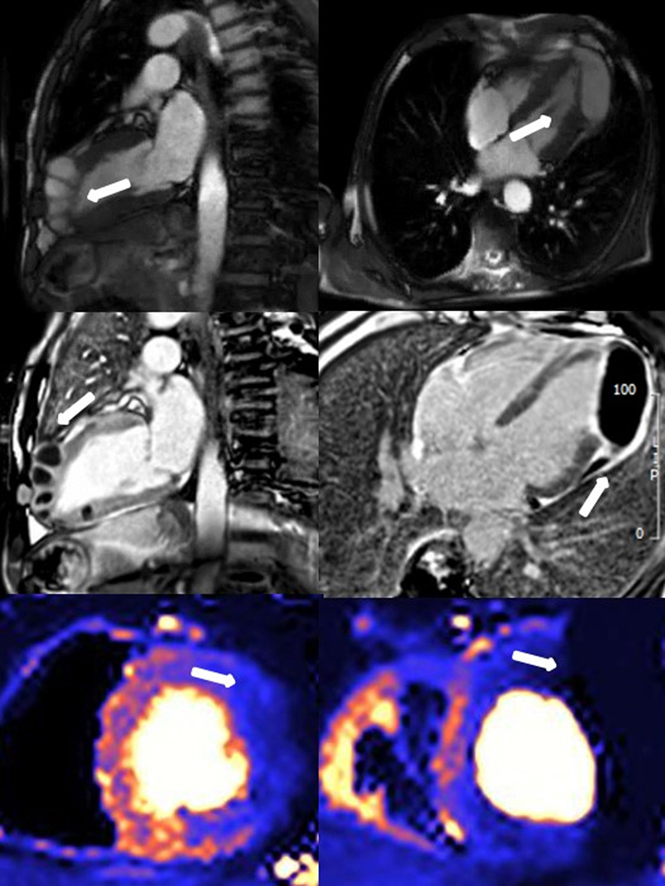

**Figure 1:**
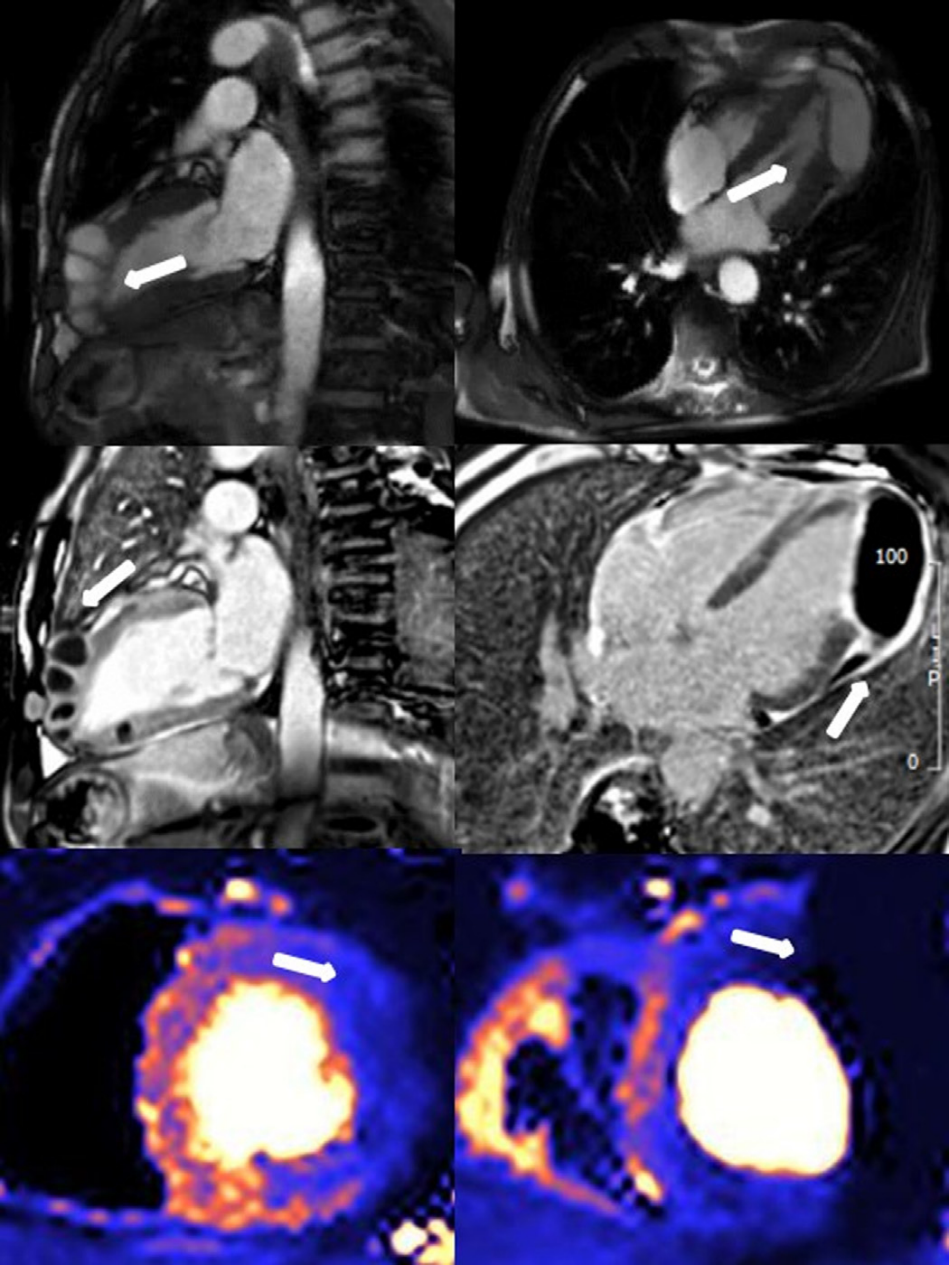
CMR Findings Apical lesions identified by the **white arrow** as follows: balance steady state procession **(top)**; late gadolinium enhancement **(middle)**; adenosine stress perfusion testing **(bottom)**.

An asymptomatic man in his early sixties underwent an elective cardiac magnetic resonance study. Minimal clinical details were provided to the reporters at the time of scanning, but the patient confirmed that he was diabetic, a current smoker, and was involved in a car accident some months before the exam. His scan demonstrated multiple apical loculated collections, with low T1 and increased T2 times, with late gadolinium enhancement surrounding these lesions. These collections did not perfuse during adenosine stress perfusion imaging. Apical hypokinesia was noted (Video 1, Figure 1), which was presumed to be secondary to the destruction of the surrounding myocardial structure. No abnormalities were detected on early gadolinium enhancement imaging, suggesting there was no direct communication between the ventricular chamber and these lesions. The initial differential for these findings was broad, including cystic echinococcosis, contained myocardial rupture, tumor, tuberculosis, or deceleration injury.

Additional information was sought regarding the reason for referral for a cardiac magnetic resonance study (Videos 2 and 3, Supplemental Figure 1). It then became apparent that this patient had previously suffered a silent myocardial infarction, with a known distal occlusion of the left anterior descending coronary artery, and the request was to determine the viability of the apical segments of the left ventricle subtended by the occluded coronary artery. With this more detailed information, the final report was extensive apical infarct with a large contained myocardial rupture.

This case exemplifies that independently of how advanced the cardiac imaging modality is, accurate and precise diagnosis remains wholly reliant on a concise yet salient history from referring clinicians to optimize the utility and correct interpretation of imaging findings.

## Funding Support and Author Disclosures

The authors have reported that they have no relationships relevant to the contents of this paper to disclose.

## Appendix

Video 1 Cardiac Magnetic Resonance Balanced Steady State Procession Images Showing Apical Intramyocardial Collections, Apical Hypokinesia, and Thinned Myocardium

Video 2 Echocardiogram: 4-Chamber Apical View

Video 3 Echocardiogram: Doppler Imaging

