# Supplementary figures and images for: Better Lucky Than Clever
Source: JACC Case Rep. 2023 Aug 10;22:101975. doi: 10.1016/j.jaccas.2023.101975 (PMC10544080; doi:10.1016/j.jaccas.2023.101975)

Supplemental Figure 1 – ECG


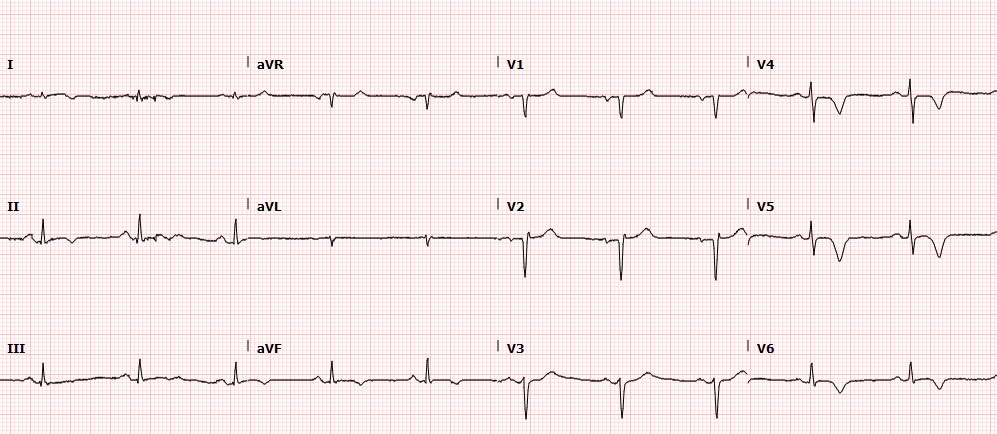

Supplement: Supplemental Figure 1 [file mmc4.docx]
